# Moderating variables of music training-induced neuroplasticity: a review and discussion

**DOI:** 10.3389/fpsyg.2013.00606

**Published:** 2013-09-09

**Authors:** Dawn L. Merrett, Isabelle Peretz, Sarah J. Wilson

**Affiliations:** ^1^Melbourne School of Psychological Sciences, The University of MelbourneMelbourne, VIC, Australia; ^2^Department of Psychology, Université de MontréalMontréal, QC, Canada

**Keywords:** music training, neuroplasticity, imaging, training age, sex, absolute pitch, training type

## Abstract

A large body of literature now exists to substantiate the long-held idea that musicians' brains differ structurally and functionally from non-musicians' brains. These differences include changes in volume, morphology, density, connectivity, and function across many regions of the brain. In addition to the extensive literature that investigates these differences cross-sectionally by comparing musicians and non-musicians, longitudinal studies have demonstrated the causal influence of music training on the brain across the lifespan. However, there is a large degree of inconsistency in the findings, with discordance between studies, laboratories, and techniques. A review of this literature highlights a number of variables that appear to moderate the relationship between music training and brain structure and function. These include age at commencement of training, sex, absolute pitch (AP), type of training, and instrument of training. These moderating variables may account for previously unexplained discrepancies in the existing literature, and we propose that future studies carefully consider research designs and methodologies that control for these variables.

In the last few decades, a considerable body of research has accrued on differences in the brains of musicians and non-musicians, as well as the changes created in the brain when becoming a musician. The findings of over 100 neuroimaging studies have been variously reviewed in a number of previous publications (Schlaug, 2001; Münte et al., 2002; Johansson, 2006; Altenmüller, 2008; Stewart, 2008; Habib and Besson, 2009; Jäncke, 2009; Tervaniemi, 2009; Kraus and Chandrasekaran, 2010; Wan and Schlaug, 2010; Herholz and Zatorre, 2012; Merrett and Wilson, 2012) and demonstrate convincingly that music has a significant impact on brain structure and function. Musicians and non-musicians' brains appear to have differences in volume, morphology, density, connectivity, and functional activity across a range of brain regions and structures. In addition to numerous cross-sectional studies, longitudinal music training studies in both children and adults have provided the most powerful evidence of music-induced neuroplasticity.

However, when the literature is sampled extensively, it becomes apparent that there are a number of contradictory findings. For example, a number of studies have looked at differences between musicians and non-musicians in the size or latency of electrical or magnetic potentials evoked in response to a variety of auditory stimuli. For some waveform components, such as the N1(m), there appear to be as many studies that have not found differences as those that have reported musician-non-musician differences (Pantev et al., 1998, 2001; Schneider et al., 2002; Schulz et al., 2003; Shahin et al., 2003, 2005; Kuriki et al., 2006; Lütkenhöner et al., 2006; Baumann et al., 2008). Another example can be seen in diffusion tensor imaging (DTI) studies of the corticospinal tract. Two studies found that fractional anisotropy (FA) in this tract was greater in musicians (Bengtsson et al., 2005; Han et al., 2009), while two other studies found that it was greater in non-musicians (Schmithorst and Wilke, 2002; Imfeld et al., 2009). Furthermore, there has been suprisingly little concordance between the results of whole-brain voxel-based morphometry (VBM) studies comparing musicians and non-musicians. Apart from the inferior frontal gyrus in the left hemisphere, no other brain regions were consistently found to be different between musicians and non-musicians across the five known VBM studies (Sluming et al., 2002; Gaser and Schlaug, 2003; Bermudez and Zatorre, 2005; Bermudez et al., 2009; Han et al., 2009). Although each study showed differences, the nature and location of these differences varied across studies. Even in the inferior frontal gyrus, which was significantly different in each of the VBM studies, the differences varied in their location on this gyrus across the anterior-posterior dimension.

These types of discrepancies have seldom been discussed in the literature to date. This could be due to the fact that until recently, many researchers were sceptical that music training would lead to differences in brain structure and function and/or were cautious in attributing causality to cross-sectional and correlational research. Previous studies and reviews have focused primarily on collating sufficient evidence that musicians' brains are different from non-musicians' brains, and that this reflects their experiences and not just their genetics. Because of the need to establish beyond doubt the very existence of music-induced neuroplasticity, these reviews have not focused on critically evaluating the concordance of the evidence. We would suggest that the field has matured to the point that this type of critical analysis is necessary to advance our understanding of music-induced neuroplasticity and to drive future research. It is clear that music training does induce changes in the brain, but there are numerous factors that influence when, where, and how neuroplasticity occurs in response to music training.

A number of variables that moderate the relationship between music training and neuroplasticity have been proposed in the literature. Our aim is to review these putative moderating variables and to discuss whether they could account for some of the discrepancies in the results of existing studies, including the examples given above. In addition, we will look at the implications of these moderators for future research designs and methodologies.

## Age at commencement of training

In 1995b, Schlaug et al., published a highly influential paper that showed that the anterior half of the corpus callosum was larger in musicians than in non-musicians, but only for those musicians who commenced music training prior to 7 years of age. Since that time, a number of additional studies have reported similar findings in the corpus callosum for early trained musicians (Öztürk et al., 2002; Lee et al., 2003). Musician-specific effects in other motor regions, such as the sensorimotor cortices and pyramidal tracts, have also been correlated with age at commencement of training (Elbert et al., 1995; Amunts et al., 1997; Li et al., 2010) or practice hours in childhood (Bengtsson et al., 2005). This fits well with the behavioral literature that shows that motor skill attainment in musicians is negatively correlated with the age at which they started training. For example, an earlier age of commencement is associated with less asymmetry in hand tapping speed (Jäncke et al., 1997). Even when total years of study are accounted for, early-trained musicians outperform later-trained musicians on motor tasks (Watanabe et al., 2007).

These findings suggest that age at commencement of training is an important moderating variable of music-induced neuroplasticity. While neuroplasticity can occur throughout the lifespan, the evidence suggests that there is a sensitive period for motor learning that music training may interact with (Penhune, 2011). Based on the findings in the literature, training commenced before age seven has become a marker for early training. Those musicians who begin training prior to age seven may show greater capacity for neuroplastic changes than those who take up an instrument later in childhood or in adulthood. However, it should be noted that these types of correlations have not been found consistently across all brain regions related to motor function or related to other sensory modalities known to be influenced by music training. Although the cerebellum is a key part of the motor system, and differences between musicians and non-musicians have been found in cerebellar volume, there does not appear to be a relationship between age at commencement of training and volume in the cerebellum (Hutchinson et al., 2003). Similarly, the results in the auditory domain are mixed, with some studies finding a correlation between age of commencement of training and measures of auditory function (Pantev et al., 1998, 2001; Trainor et al., 1999; Wong et al., 2007; Musacchia et al., 2008), while another study looking at the structure of auditory cortex showed no difference (Keenan et al., 2001).

Given that this variable has not been accounted for in many studies comparing musicians and non-musicians, its impact is not fully understood. However, it could possibly account for some of the discordant findings in the literature. For example, in studies looking at FA of the corpus callosum, one study that used early-trained musicians found musician-non-musicians differences (Schmithorst and Wilke, 2002), while other studies that included musicians who commenced training after aged seven did not (Han et al., 2009; Imfeld et al., 2009). While this is a highly plausible explanation in light of the existing literature, other explanations could include differences in imaging acquisition and analysis, as the specific techniques used in these studies can have a significant impact on the results (Jones, 2010). Thus, while accounting for age at commencement of training may not solve all the existing discrepancies, it is clear that it has a significant effect on outcomes and should be reported and analyzed in future research. It might also interact with other moderating variables, such as sex and absolute pitch (AP) ability, which are discussed in more detail below.

## Training and practice parameters

Our understanding of music-induced neuroplasticity can be advanced by considering not just when musicians start to train, but how long and how much they train. Some of the studies mentioned above that did not find correlations between age at commencement of training and structural or functional neuroplasticity in musicians did find a relationship between the amount, duration, or intensity of practice and neuroplastic changes. For example, in the study by Hutchinson et al. (2003), lifetime intensity of practice was correlated to cerebellar volume even though age at commencement of training was not. A longitudinal study investigating the influence of music training on brain structure in children found a significant relationship between the amount of practice and the degree of structural change in the corpus callosum (Schlaug et al., 2005), while another study found that practice hours in childhood, adolescence, and adulthood were correlated with FA of various white matter tracts (Bengtsson et al., 2005). In the auditory domain, several studies have found a correlation between brainstem encoding of music and speech sounds and years of continuous training and/or amount of practice (Musacchia et al., 2007, 2008; Wong et al., 2007). In a behavioral study, Wilson et al. (2012) have shown that ongoing music engagement (current hours spent practicing per week) influences the accuracy of AP ability, even though this skill was once thought to be entirely determined by genetics and early music training. These studies clarify that not only early training, but accumulated practice and recency of practice may be moderating variables of music-induced neuroplasticity.

This raises questions about the stability of training-induced neuroplastic changes, and whether ongoing training is required to maintain such changes. Would significant structural changes induced by early training during sensitive periods remain even if musical training ceased shortly thereafter? In other words, is there a point at which neuroplasticity becomes fixed? Studies outside of the music domain in adults have suggested that structural changes induced by a complex motor task (e.g., juggling) occur within 1 week of training, but return to baseline without ongoing training (Draganski et al., 2004; Driemeyer et al., 2008). These studies also suggest that it is the act of learning the task that induces neuroplasticity, not ongoing practice or maintenance of the task. Driemeyer et al. (2008) found that juggling training led to neuroplastic changes within the first 7 days, but ongoing practice over another month (with concomitant skill improvement) did not create further changes. This indicates that there may be a difference in outcomes between paradigms that focus on training new tasks vs. those that focus on repeated practice of learned tasks. Although the terms training and practice are often used interchangeably in the literature, we propose that these terms could be differentiated to indicate whether a learning paradigm includes novel, challenging tasks with corrective feedback (training) or repetition without external feedback (practice). There may be important neurobiological differences between “training” and “practice,” and thus, the two terms should not be used interchangeably.

Most of the music-induced neuroplasticity literature has not accounted for current music engagement, the recency, intensity, and complexity of training, the extent of “training” vs. “practice,” and other related variables. Given that such training and practice variables have been linked to the degree of both structural and functional changes in musicians, they should be accounted for in comparison studies between musicians and non-musicians.

## Instrument and type of music training

In addition to when, how long, and how much training occurs, both the type of instrument and the type of training may influence neuroplasticity in musicians. In a simple experiment, Bangert and Schlaug (2006) showed that pianists, violinists, and non-musicians could be differentiated by the morphology of their motor cortex. Pantev et al. (2001) found that functional auditory responses were greatest to a musician's own instrument, with musicians demonstrating timbral specificity to their training background. A related finding by Gebel et al. (2013) indicated that trumpeters had greater functional activation in the cerebellum, dominant sensorimotor cortex, and left auditory cortex than pianists when performing trumpet-related activities. Tervaniemi et al. (2001) suggested that musicians who played by ear and improvised could learn to process complex musical information more accurately than classically trained musicians, with corresponding differences in auditory neural responses.

Many researchers have questioned whether these types of cross-sectional studies provide adequate evidence for training-induced neuroplasticity, since pre-existing genetically-modulated differences in brain structure and function could account for these results (see below for additional discussion regarding pre-dispositions and genetics). Individuals who already have certain brain characteristics might choose particular instruments or types of training. Given the specificity of the effects, however, a more likely explanation is that the demands of individual instruments or types of training lead to differential neuroplasticity. This is supported by a longitudinal study in children in which there were no group differences in brain structure observed prior to training in those intending to study either piano or violin and those not intending to study music (Norton et al., 2005).

If different instruments of training can lead to striking structural differences, this could help explain why VBM studies comparing the brains of musicians and non-musicians have not been more concordant, as mentioned above. Across the five VBM studies to date, each has used different musician groups, with some using only pianists and string players, and others using mixed groups (orchestra-based musicians). It could also explain contradictory findings in DTI studies looking at the corticospinal tract. For example, the two studies that showed increased FA in musicians used pianists (Bengtsson et al., 2005; Han et al., 2009), whereas two studies that showed decreased FA in musicians used mixed groups of musicians (Schmithorst and Wilke, 2002; Imfeld et al., 2009). While there are other possible explanations, such as methodological differences in the acquisition and analysis of the diffusion tensor images, this provides a reasonable hypothesis as to why contradictory results have occurred.

## Sex differences in the brain

Differences between males and females in brain structure and function have been well-described in the literature (for example Shaywitz et al., 1995; Good et al., 2001; Gaab et al., 2003; Koelsch et al., 2003). These differences include a greater degree of overall symmetry and less hemispheric specialization in the female brain, as well as gender specific asymmetries in particular regions and structures. Although still a controversial hypothesis, it appears that sex may also modulate neuroplastic capacity. This may be due to differences in sex hormones as well as genetic effects (Ngun et al., 2011). A number of studies have found differences between males and females in the induction or degree of neuroplasticity, such as heightened cortical excitability after anodal transcranial direct current stimulation (tDCS; Chaieb et al., 2008) and greater inhibition following cathodal tDCS (Kuo et al., 2006) in females. However, when considered more closely, these studies contradict each other (Chaieb et al., 2008 did not find a significant sex difference after cathodal stimulation, while Kuo et al. (2006) did not find a difference after anodal stimulation). Moreover, other studies have failed to find any sex differences in neuroplasticity (Sale et al., 2008), indicating that our understanding of this variable is incomplete. Joel (2011) suggests that sex interacts with environmental factors in complex ways that ultimately influence brain structure and function. This could certainly hold in the case of music training-induced neuroplasticity, leading to a moderating effect of sex on the type, location, and degree of neuroplasticity observed in response to music training.

Several studies comparing musicians and non-musicians' brains have found a difference between male musicians and non-musicians, but not between females. For example, as a follow-up to the 1995 corpus callosum paper by Schlaug and colleagues, Lee et al. (2003) reported that there was an effect of musicianship on the corpus callosum only in males. These researchers hypothesized that this may reflect pre-existing sex-based differences in brain symmetry or the influence of AP on brain structure, which was overrepresented in their female musician group. Using VBM, Luders et al. (2004) also reported significant gender and AP effects on gray matter asymmetries in musicians, with increased leftward asymmetry of the postcentral gyrus of male non-AP musicians compared to female non-AP musicians, as well as interactive effects of sex and AP on the localization of asymmetry along the superior temporal gyrus. In another structural study, only male musicians were shown to have greater cerebellar volumes than non-musicians, with no significant difference in females (Hutchinson et al., 2003).

These results and concerns about sex as a moderating variable have led to an interesting phenomenon in the literature, in which a number of studies comparing musicians and non-musicians have only used males (for example, Amunts et al., 1997; Gaser and Schlaug, 2003). However, other studies have used musician groups with a large proportion of females, and their significant results suggest that structural and functional brain differences can be observed in female musicians (for example, Bermudez et al., 2009; Han et al., 2009). For this reason, it is clear that specific effects of sex on plasticity require further investigation to understand how, when, and where sex modulates the effects of music training on brain structure and function. Interpretation of current research in this field is complicated by the observation that many previous studies have not obtained large enough or appropriately balanced samples in which sex could be included as a variable.

## Absolute pitch

AP, the ability to correctly label or produce pitches without a reference pitch, has been linked with specific structural and functional brain differences. Musicians who possess this skill appear to have increased volume of Heschl's gyrus in the right hemisphere (Wengenroth et al., 2013), leftward asymmetry of the planum temporale (Schlaug et al., 1995a; Zatorre et al., 1998; Keenan et al., 2001; Wilson et al., 2009), reduced thickness of the dorsal frontal cortices (Bermudez et al., 2009), and leftward asymmetry of FA in the superior longitudinal fasciculus (Oechslin et al., 2010). They also process pitch, timbre, and intervals differently than other musicians as well as non-musicians (Klein et al., 1984; Hantz et al., 1992; Wayman et al., 1992; Crummer et al., 1994; Zatorre et al., 1998; Ohnishi et al., 2001; Wilson et al., 2009). Although the emergence of AP is thought to be dependent on early music training (Zatorre, 2003; Levitin and Rogers, 2005) and recent research suggests that it is linked to ongoing music practice (Wilson et al., 2012), the structural and functional brain changes appear to be distinct from those due to music training alone. The level of AP skill also impacts on structural and functional differences, with those musicians who have the ability to label a limited number of pitches without a reference (quasi-AP) showing more symmetry of the planum temporale than either AP or non-AP musicians (Wilson et al., 2009).

Given the widespread influence that AP appears to have on the brains of musicians, this variable could confound the measurement of training-induced neuroplasticity in studies in which it is not taken into account. Unfortunately, a fairly large proportion of the literature comparing musicians and non-musicians' brains has not reported whether their musician sample includes individuals with AP. In some cases, it has been offered as a potential *post-hoc* explanation without being accounted for in the data analysis. The possibility of interactions between AP and other moderating variables should also be considered. For example, Luders et al. (2004) reported differences in the location of asymmetry in male and female musicians, with male AP musicians showing leftward asymmetry of the planum temporale and female AP musicians showing leftward asymmetry within Heshl's gyri. The effects of AP on brain structure and function could interact with variables such as age at commencement of training, type of training, and instrument of training, since AP expression is more common with early training, training using a fixed-do system, and training on fixed pitch instruments such as the piano (Wilson et al., 2012). AP expression is also thought to depend on genetic predisposition (Theusch et al., 2009; Theusch and Gitschier, 2011), thereby raising issues of individual differences and genetic influences on brain structure and function, which we turn to next.

## Genetics, environment, and individual differences

In addition to discrepant findings between studies, some music-related training studies have reported discrepancies in the neuroplasticity outcomes of training within their own participant samples. For example, in two auditory training studies, one looking at pitch deviant detection and the other at pitch working memory skills, both found a subset of participants who made behavioral improvements and showed related neuroplastic changes and another subset of participants who did not show the same degree of improvement or related neuroplasticity (Jäncke et al., 2001; Gaab et al., 2006). An early motor training study by Schlaug et al. (1994) found that the spatial coordinates of neuroplastic change, which were related to movement accuracy and speed, did not show much overlap between participants and were therefore not evident in group level analysis. Although it is unclear whether a large degree of intersubject variability has led to null results in other music neuroplasticity studies, it seems highly likely. The causes of these individual differences are presumably both genetic and environmental.

Certain aspects of music ability, such as AP (Zatorre, 2003), difficulty processing pitch (congenital amusia; Peretz et al., 2007), and even musicality or sensitivity to music (Martens et al., 2008; Wengenroth et al., 2010), have been linked to genetic factors. While genetic studies of music abilities are still ongoing (for example, Park et al., 2012; Ukkola-Vuoti et al., 2013; for a comprehensive review, see Tan et al., submitted), it is clear that genes play a role. Recent evidence suggests that training-induced neuroplasticity may not account for all of the anatomical and behavioral variability observed in expert brains, and that pre-existing (likely genetic) anatomical features and behavioral skills may be further modified by training (Foster and Zatorre, 2010; Golestani et al., 2011). Moreover, genes can influence music ability and music-induced neuroplasticity apart from specific music skills through more general neurocognitive capacities that are genetically modulated and are involved in, but not specific to, music learning (Friedman et al., 2008; Frank and Fossella, 2011; Ukkola-Vuoti et al., 2013). In his models of giftedness and talent, Gagné (1999) maintains that practice and other environmental factors always operate on natural abilities with a genetic basis. Great emphasis has been placed in the literature on the role of accumulated deliberate practice in expert musicianship (Ericsson, 2006), but genetic influences should not be discounted. Learning capacity and even the motivation to engage in training/practice may be genetically modulated (reviewed in Frank and Fossella, 2011), thereby leading to differences in outcomes between those with similar or standardized training. For example, personality variables, which are in part genetically determined, have been implicated in the choice to take music lessons and also the extent of engagement (duration of music training; see Corrigall et al., 2013; this issue).

Even before music training occurs, environmental differences could play a role in future training-induced neuroplasticity. For example, a study in preschool children by Shahin et al. (2004) suggested that early music exposure (such as another musician in the home) might be related to functional auditory differences that were already evident prior to training. In addition, a number of studies now suggest that the phenomenon of metaplasticity interacts with music-induced neuroplasticity, with musicians' brains seemingly more capable of neuroplastic change (Tervaniemi et al., 2001; Ragert et al., 2004; Rosenkranz et al., 2007; Herholz et al., 2011; Seppanen et al., 2012). Metaplasticity is defined by Abraham (2008) as activity-dependent mechanisms that regulate the expression of future plasticity at both individual synapses and the network level. One could say that plasticity begets plasticity. Since previous music training influences subsequent neuroplasticity, it is not far-fetched to assume that previous training in other (perhaps related) fields could affect music training-induced neuroplasticity, providing another potential explanation for individual differences in previous studies.

## Study design, analysis, and interpretation

While we propose that the above-mentioned variables may provide at least a partial explanation for the lack of consistency in music training studies, we also acknowledge that study design and methodology could play a large role. For some discrepancies in the literature, no specific moderating variable discussed in this review presents itself as a more likely explanation given the available information. For example, the variation in results for studies of N1 evoked responses, in which half of the studies show differences between musicians and non-musicians and the other half do not show differences, are not yet understood. While moderating variables could be contributing to these discordant outcomes, methodological differences may also account for the variability. These studies, and the literature as a whole, show great diversity in both behavioral task parameters (used to quantify music skill) and in brain imaging acquisition and analysis techniques. For instance, small differences in stimuli, such as intensity or other stimulus features, or differences in the way the stimuli are presented, such as the salience of the distractor for non-attended auditory paradigms, can lead to differences in how the brain responds during functional studies (Martin et al., 2007).

As mentioned previously, the results of DTI studies and other investigations of white matter and gray matter are also significantly influenced by the imaging methodology employed (Jones, 2010). Finding significance can be dependent on whether a study looks at the whole brain or at smaller *a priori* regions of interest, as significance is highly impacted by the chosen method of dealing with the multiple comparison problem in imaging. More generally, statistical power issues are common in this field of research, particularly in fMRI and VBM studies, due to the costs of imaging. Many studies can be found in this literature with fewer than 10 participants, leading to difficulties with reproducibility and interpretation given well-recognized individual differences in brain anatomy and function.

Another important factor to consider is the diverse backgrounds and preconceptions that researchers bring to their work. This can result in the use of different operational definitions for variables and biases in the interpretation of results. For example, the definitions of “musician” and “non-musician” vary from study to study, with non-musicians having between 0 and 3 years of training across the studies sampled. Given that a longitudinal study in children showed structural changes evident after just 1 year of training (Hyde et al., 2009), it is probable that “non-musicians” with short-term training (for example, 1–3 years) that occurred in early childhood have already experienced some music training-related neuroplasticity. In short, over and above any participant variables, the study design itself, the operational definitions used, and interpretational biases are likely to have a significant impact on results.

## Conclusions

Given the large number of variables listed here, which may account for discrepancies between music training studies, the concordance that *is* observed across studies makes the evidence for music-induced neuroplasticity very compelling. The purpose of highlighting these differences and the variables that may underlie them is not to negate previous work in this field, but to push it forward and spark a critical examination of study designs and methodologies for future research. The role of these moderating variables should be systematically examined in future research, and also accounted for, or at the very least reported, when they are not the primary variables of interest. This allows such research to be more useful across domains, as it can play a significant role in understanding not just music neuroscience but also brain function and neuroplasticity more generally.

**Figure 1:**
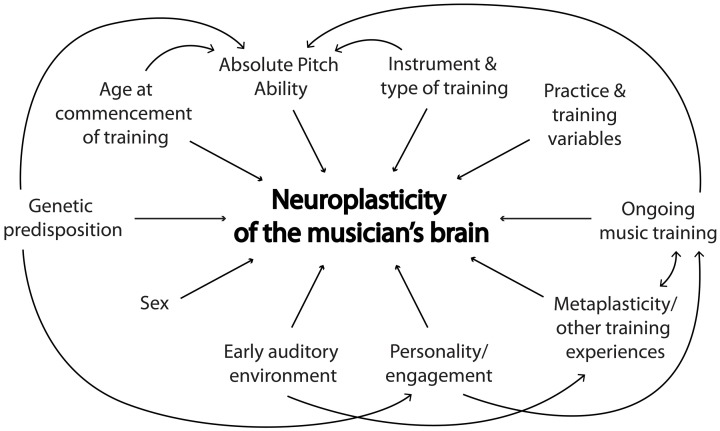
**A schematic representation of moderating variables of music-induced neuroplasticity and their interactions**.

The current trend toward longitudinal training studies, whereby most subject- and training-related variables can be more easily controlled, is an important step forward in music neuroscience. Prospective longitudinal designs are optimal to control for putative moderators such as age at commencement of training, instrument and type of training, and duration, recency and intensity of training. The error variance associated with subject-related variables such as sex, AP ability, genetics, and environmental factors is also reduced in within-subject designs. While cross-sectional designs comparing musicians and non-musicians are more time- and cost-efficient than longitudinal studies, they lack experimental control as well as the capacity to test hypotheses about causality. For such reasons, we would suggest that future studies consider longitudinal designs wherever feasible. Corrigall et al. (2013) raise some significant difficulties that arise with longitudinal training designs, such as funding concerns, differential attrition, and lack of engagement in protocols. While we acknowledge these issues, we believe that longitudinal studies offer the best chance to tease apart the relative contributions of potential moderating variables, and thus, understand the formation of the musician's brain (See Figure 1).

Far from being nuisance variables, each of the variables discussed has already provided rich information about brain plasticity. Ideally, new music training research that controls and also manipulates these variables will continue to provide fresh insights. Although this presents a challenge, it is not insurmountable. For instance, imaging acquisition and analysis techniques are constantly evolving, but it is hoped that as gold standard techniques emerge, they will be widely adopted. More detailed reporting of imaging protocols and analysis will also allow for better replication and comparison between studies. Despite the difficulties in accounting for some of the variables, we also hope to see ongoing improvements in the ability to assess genetic predispositions, early environmental factors, and level of engagement in training. The way that these factors influence training outcomes and the way they come together within different individuals are likely to be increasingly important as we tackle how and why music shapes the brain.

### Conflict of interest statement

The authors declare that the research was conducted in the absence of any commercial or financial relationships that could be construed as a potential conflict of interest.
